# Exploring the evolution of a dental code of ethics: a critical discourse analysis

**DOI:** 10.1186/s12910-020-00485-3

**Published:** 2020-06-03

**Authors:** Alexander C. L. Holden

**Affiliations:** Faculty of Medicine and Health, The University of Sydney School of Dentistry, 2-18 Chalmers Street, Surry Hills, NSW 2010 Australia

**Keywords:** Critical discourse analysis, Dentistry, Code of ethics, Professionalism, Sociology

## Abstract

**Background:**

What can the analysis of the evolution of a code of ethics tell us about the dental profession and the association that develops it? The establishment of codes of ethics are foundational events in the social history of a profession. Within these documents it is possible to find statements of values and culture that serve a variety of purposes. Codes of ethics in dentistry have not frequently presented as the subjects of analyses despite containing rich information about the priorities and anxieties within the profession’s membership at the time that the code was written.

**Main text:**

This essay uses critical discourse analysis to explore the 2012 and 2018 versions of the Code of Ethics produced by the New South Wales Branch of the Australian Dental Association. This method of discourse analysis examines contradictions between the discourses within the codes and how these relate to broader social realties that surround the dental profession in New South Wales. By analysing the 2012 and 2018 codes together, it is possible to understand how the dental profession views its commitments to society as established through the social contract. Through this assessment, it will be demonstrated that both codes suffer due to their failure to consider the public as a key stakeholder in the creation and curation of the Code of Ethics and how this this relates intimately with the social contract between the profession and the public.

**Conclusion:**

Without the public being the central consideration, both codes amount to declarations of professional privilege and dominance. Although the more recent 2018 Code of Ethics demonstrates insight into the changes in public trust placed in the professions, this analysis shows that that the current code of ethics is still reluctant to recognise and engage with the public as an equal stakeholder in the planning and provision of oral health care and the development of the profession’s values and cultural trajectory.

## Background

Through a complex interaction of social, economic, legal and political events, the public’s expectation of professionals has changed. The long-enjoyed trust, respect and privilege gifted to those with professional status, whilst still present, has been eroded. The public increasingly demand equality and accountability from professional groups and their members. Social contract theory as applied to the health professions references the system of tacit promises and obligations that exist, both in written and unwritten form, between society and professional groups. The written and unwritten nature of the social contract is illustrated through examining the nature of its different facets. The obligation for professionals to engage in self-regulation has become more clearly defined through statute and precedent in the courts, whilst the obligation for health professionals to provide care in a manner which effaces their own self-interest is still embodied as more of a social and professional expectation. The social contract between professionals and society grants privilege on the premise that the professions will enhance social well-being and behave in a manner which is consistent with key expectations [1, 2]. The most important expectation that health professionals must meet, is to ensure the effacement of self-interest in placing the interests of patients first [3]. The commercialisation of healthcare, especially dentistry, has contributed greatly to the tension between self-interest and ethical obligations [4, 5]. The social contract in dentistry has been keenly discussed as to whether cosmetically-driven treatments fall within the professional purpose, and therefore the social contract of dentistry [6, 7]. The nature of the social contract in dentistry is suggested to be developing, with several theorists arguing that the social contract in dentistry is at risk due to issues relating to lack of engagement with self-regulation, the commercialisation of care and inequity in access to dental services [8, 9]. Therefore, it has never been more important for the code of ethics of a dental professional association to reflect their position as a moral community, reflecting that service to the public is the principal purpose for the profession’s continuation as a privileged group of skilled practitioners.

In a group of professionals, the next generation is not produced biologically, but socially and culturally [10]. The moral community formed by a professional organisation should ensure that the culture and social environment is able to foster the development of natal professional members so that they are able to meet the key expectations of the social contract. Pellegrino and Relman recognised the importance of professional associations acting as moral communities; “Professional associations exist to proclaim, protect, refine, teach, and enforce that behaviour. Without such a commitment, they easily degenerate into self-serving trade associations, lobbies or unions.” [11] They suggested that many professional associations within medicine have already begun to act as corporatized bodies that act with the interests of members as prime consideration. Pellegrino [12] described professional associations as moral communities, being responsible for supporting the medical profession against commercial pressures that transform healthcare into a marketplace. This vision of professional communities is Pellegrino’s statement of ideal. Healthcare professions have always been driven, in part, by self-interest and this is frequently reflected within codes of ethics and conduct. In the historic example provided by the inaugural code of the American Medical Association published 1847, the code appears as a document designed to protect the monopoly of the medical profession, through the prevention of conduct such as providing discounted or pro bono treatments and the prohibition of advertising [13]. The later equivalent produced by the American Dental Association in 1866 espoused similar ideals, with professional conduct being synonymous with gentlemanly conduct [14].

It is a traditional requirement that professions have codes of ethics or conduct. The nomenclature of these typically reflects how each profession might choose to use a code. Frankel describes three types of code; aspirational, educational and regulatory [15]. In New South Wales, Australia, the conduct, performance and health standard of the dental profession is dictated by the Code of Conduct that has been developed collaboratively by several of the National Boards, including the Dental Board of Australia [16]. This code is prescriptive and sets out expectations relating to health, conduct and performance. The Code of Conduct is frequently used to determine whether practitioners have breached accepted standards of professional conduct and therefore falls into the category of being a regulatory code. Where a professional association has a code of ethics that exists alongside a regulatory code of conduct, the association should consider its code of ethics to hold a different purpose. An aspirational code is a statement of ideal behaviour that practitioners should seek to publicly demonstrate. A code may also be educational, where commentary and elaboration help practitioners to understand how the code might assist them in navigating ethical challenges associated with the profession. Other commentators have noted that producing a code fulfilling this purpose is challenging; codes may be critiqued for being too vague to be applicable to be useful in guiding practitioners in real-life situations [17].

In April 2018, the NSW Branch of the Australian Dental Association (ADA NSW) released an updated Code of Ethics [18]. This concluded a process of internal review where the previous version of the code was updated [19]. The internal review involved consultation within the ADA NSW; there was no public or external (non-professional) involvement in the review of the Code of Ethics. The ADA NSW is a state branch of the Australian Dental Association which is the peak professional body in dentistry in Australia. At a reported 4595 members [20], the NSW branch is the largest in Australia. This paper analyses the evolution of the code from 2012 to the updated version published in 2018 and explores how changes might impact upon the nexus between the dental profession in NSW and society. Examining the codes will be done through the theoretical framework of the social contract, first described in the context of medicine by Starr [21] and developed by Cruess and Cruess [2]. To summarise this framework in the context of dentistry, the social contract is an exchange of promises and commitments made between society and the dental profession. Society is reliant on the skills of the profession for the relief of pain and suffering [6] as well as reasonable aesthetic enhancement [7]. The expectation is that practical skills will be provided altruistically, with competency and trust that the profession will engage in self-regulation [8]. In exchange, society provides the dental profession with higher social status, recognition of a professional monopoly, professional prestige and appropriate remuneration. The principles of the social contract also demand that the dental profession, including dental professional associations, take action against commercial influences which do not promote the interests of patients and address inequitable access to oral health services for vulnerable groups within society.

In both the codes that will be analysed, the place of the voice of the public and patients will be considered, as well as how the code embodies the relationship between the profession and society set out by the social contract.

## Main Text

### Methods

To develop understanding of how the Code of Ethics has evolved between the 2012 and 2018 versions of the code, a critical discourse analysis was carried out.

Critical discourse analysis provides a means to explore relationships between discursive texts, events and practices and wider social and cultural structures, relations and processes. This will allow investigation to how the codes of ethics are shaped by power relations that form between the dental profession and various stakeholders. These relationships can be viewed as having opacity; those involved within these interactions may be unaware of the linkages between ideology, power and discourse [22].

Fairclough states; “our chances of changing existing social reality for the better in part depend upon understanding it better, and that includes understanding how relations between discourse and other elements of it contribute to the way it works.” [23] (page 46) Fairclough describes critical discourse analysis of having three interconnected parts or stages; 1) normative critique of discourse, which leads to; 2) explanatory critique of discourse; and 3) transformative action which positively affects the existing paradigm.

Normative critique identifies contradictions within social realities. In other words, the purpose of this part of the analysis is to distinguish between what is said to happen, and what actually occurs within a particular social reality. In the context of this exploration of the Codes of Ethics, this means that where a statement is made, the accompanying features within the code such as the language used, its position and the orientation of what is being professed may create a contradiction between the message and the discourse surrounding it. Contradiction has previously been identified within professional codes by Gibb; “Not all the planks of a professional association’s code of ethics are meant to be taken in the same spirit. Some are merely costumes the profession puts on to impress outsiders. Some are preachments to be honoured, but not necessarily obeyed. Some are guides, but permissive ones. Some are tactical mover in controversies with outside groups. Some are really seriously intended.” [24] (page 242) This analysis will examine and critique contradictions where they arise in the ADA NSW codes.

Explanatory critique acts as the link between normative critique and action. When contradictions are identified through normative critique, if they can be explained as an effect of certain features of an existing social order which is flawed, taking action to correct this is an appropriate and socially responsible reaction [25]. Fairclough emphasises that there is a shift in what is being critiqued between the two initial stages; in normative critique it is the discourse being examined, whilst in explanatory critique it is the social reality within which the discourse exists [23].

### Limitations

It is important that the descriptions of the discourses within this analysis are not taken as having faithfulness to the external world, but as perspectives of the social and cultural milieu that require critical analysis [26]. This analysis is a qualitative interpretation that calls upon the researcher’s own ideological evaluation and as such, this work seeks to be persuasive rather than claiming universal truth. In the same way as Simpson and Mayr articulate, this analysis hopes to encourage the dental profession to further reflect upon the discourses that surround and influence the narrative of the Code of Ethics of the ADA NSW and how these impact upon the profession’s relationship with society [27].

### Normative critique

Both the 2012 and the 2018 incarnations of the ADA NSW Code of Ethics exist as codes which accompany a disciplinary process. The 2012 code includes the disciplinary procedures within the code, the 2018 code is presented as a stand-alone document distinct from any formal procedures designed to deal with transgressors. This perhaps represents the reality that, as a professional organisation dependent upon the fees of individuals who have no obligation remain as members; the ADA NSW is unlikely to engage in disciplinary proceedings in earnest, except in the most grave of circumstances and member misdemeanours. This is supported by the statement within the introduction of each code; “If Members breach this Code, they may be required to answer a complaint brought against them.” The use of the passive term, “may” suggests that the organisation wishes to maintain its right to discipline members, but this is not assured. In both codes, it is stated that where matters of conduct are covered by statutory or regulatory provisions, these override the authority of the code. This is suggestive of a misalignment existing between the standards within the ADA NSW Code of Ethics and those of relevant regulators. This also reflects the conflict between the mission of regulators who are tasked with the protection of the public, and the professional association that supports the interests of its professional members. Ideologically speaking, these should amount to a significant alignment, with professional’s first care being to the interests of the patient (the public). However, and as this analysis demonstrates, the primary tenet of professional duty does not hold prime place within the examined codes.

The introductory section of both versions of the code state that the code does not seek to act as an exhaustive list of situations and circumstances and that members should act within the “spirit” of the code. This would seem to conflict with the apparent purpose of the 2012 code where it is combined with a set of disciplinary procedures. Whilst this association is distanced in the 2018 code, the warning against non-compliance with the code, and the consequences of transgression, persists. Both versions of the code appear to hold elements of being both aspirational and disciplinary in nature.

Both codes include a section outlining Beauchamp and Childress’ four principles of biomedical ethics [28]. The codes espouse the dental profession’s commitment to serving the community ahead of personal or sectional interests. How well the codes follow this profession of self-effacement will form much of the following critique. Both codes would appear to enshrine the dental profession’s authority in the discussion of autonomy; “Dentists need to be sensitive to an individual’s personal preferences in deciding about health care, so that recognising when patients require dentists to make their decisions for them is of equal importance with respecting a patient’s treatment choice.” Despite this comment, the codes move on to state that; “dentists deliver empowerment to their patients.” The dentist’s role as a figure of authority is further affirmed by the codes in their identical discussion of the principle of justice; “Dentists must also apply the principle of justice when deciding whether to offer or withhold treatment based on personal lifestyle or health choices our patients make.” This speaks to the dentist’s position as the most powerful partner in the treatment relationship. It is also suggestive of a strong belief that oral health issues are a matter of personal responsibility; the dentist’s role being to ‘judge’ patient’s behaviours relating to diet and oral hygiene and make decisions on whether patients deserve treatment or not. Neo-liberal attitudes within the dental profession have been discussed previously in an analysis examining discourses in media sources featuring the profession and dentistry [29]. The question of whether dentists see value in discussing ethical issues in clinical practice is answered in part by a statement made in relation to confidentiality and privacy; “While dentists face far fewer moral conflicts than other health care professions in offering and carrying out treatment for their patients, as health care professionals we observe law relating to confidentiality and balance these with a requirement to report certain suspicions or confirmed instances where patients have broken the law.” This latter statement has been removed from the 2018 version of the code, the previous iteration holds that acting in accordance with the law and in an ethical manner to be synonymous with one another.

The rest of the codes are split into sections that outline particular ethical responsibilities to different parties. The 2012 code lists these as: obligations to patients; obligations to employees; obligations to other members; obligations to the dental profession; and obligations to the ADA NSW. The 2018 code differs subtly but significantly, with obligations to employees changing to obligations in employment and adding an additional section listing obligations to the community.

The 2012 code makes some noticeable statements that enshrine the professional power of the dental profession. Within the section listing obligations to patients, the statement is made; “Members are entitled to refuse any patient for treatment.” This is modified by a follow-up statement stating the members are obliged to comply with anti-discrimination legislation. These sentiments are displayed in the 2018 code, but not given in such a stark or brash manner; “Except where they would be failing in their duties on humanitarian grounds, Members have a right to decline to treat a patient provided the reason for refusal does not contravene any legislation or principle of law.” The 2018 code sees the addition of a statement relating to the provision of excessive or unnecessary treatment. This statement would seem to support the code’s function as a disciplinary tool, giving specific scenarios as actionable offences, rather than as an aspirational code. Both codes frequently treat professional ethics and the law as being synonymous with one another.

Another significant change in the tone of the Code of Ethics when contrasting the 2018 version with its predecessor, is the apparent change in rhetoric in how members might raise concerns relating to other practitioners. The National Law that regulates the dental profession in Australia mandates reporting in certain situations. Neither code makes explicit reference to this, despite referencing other statutory and legal obligations and the rights of the dental profession. The 2012 code does not encourage members to report concerns about colleagues; “Where a patient seeks an opinion from a Member … the Member consulted while observing the health and safety of the patient as their first duty, shall not call into question the professional integrity of their established dentist.” The 2018 code removes this statement, instead replacing it with a statement that encourages open and respectful communication about colleagues. The exact meaning of this in relation to raising concerns and self-regulation is difficult to ascertain, especially when the 2018 code seems to reference the previous statement in the 2012 code at a later point; “Members should always uphold and enhance the integrity and dignity of the profession.” The 2018 code encourages members to give opinions in an “objective” manner. Discussion of objective approaches to raising concerns raises that objectivity is problematic in that it may amount to sophistry given that dentistry has both objective and subjective elements due to existing as both an art and a science [30, 31].

The 2018 code sees a change from obligations to employees to obligations in employment. This is in recognition of the increase in size and number of corporate entities within dentistry. Concerns regarding perceived infringement of clinical autonomy and increased competition are apparent within the code. This is shown by statements such as; “Members should refrain from entering into any contract with a colleague or organisation which they consider may conflict with their professional autonomy, clinical independence or primary obligation to the patient.” and; “Members have an obligation to observe the legal requirements of both Employment and Competition law.” Whilst the interests of the patient are again evoked, the structure and surrounding context of the statements suggests a desire to protect the interests of dentists as a primary motivation of this statement.

The 2018 code continues to promote the professional dominance of dentists within the dental profession through statements about supervising “auxiliaries”. The legal requirement for the supervision of dental hygienists, dental therapists and oral health therapists was removed in 2013. The use of the term “auxiliaries” to describe these team members is widely considered to be derogatory. Through this discourse, the codes promote the hegemony of dentists within the dental profession.

The final point within this normative critique relates to justice. Within the new section on obligations to the community included in the 2018 code, statements are made that affirm that dentists should contribute to the oral health of the community, as well as endeavouring to improve quality of, and access to dental services. There is no mention of how dentists might accomplish this. There is also no discussion on how, in a system where the majority of dental treatment is provided privately, oral health inequities might be addressed, or whether there is a positive individual or collective duty for members of the ADA NSW to do so.

### Explanatory critique and transformative action

This element of critical discourse analysis involves stepping away from a pure assessment of the discursive elements within the texts, to think about the social reality that the discourse exists as a part of and is developed from. How the existing social reality has shaped the discourse within the codes of conduct is an important consideration if this analysis is to progress to consider how transformative action might be brought about. The analysed versions of the ADA NSW Code of Ethics are valuable in developing insights into the existing social reality; the codes are potentially the most explicit and visible pronouncement of what might be considered as the professional norms that exist within the dental profession. Whilst the ADA NSW Code of Ethics might not represent the voice of all within the profession, this text still represents the authority of the collective consciousness of the dental profession in NSW, especially those within the organisation who participated in the development of the codes. It is in effect, the profession’s testimony to its moral dimension [12]. The values that are set-out in the code provide insights as to the profession’s interpretation of the social reality they exist within. Despite codes of ethics acting as a public statement of intent, it has been argued that following a code of ethics diminishes the ability of a professional to act as an individual agent, rather than a professional agent following pre-established rules rather than responding to the needs of the individual patient [32]. Future versions of the code might reconsider their purpose as being truly aspirational, in doing so, avoiding the current tendency to be prescriptive with the code serving to dictate professional norms of behaviour.

The ADA NSW Code of Conduct similarly follows a general trend of dental codes of ethics showing clear unease around the subject area of reporting professional colleagues who fall short of standards of health, conduct or performance [31]. Within the 2012 code, reporting is not mentioned in the context of protecting the public. The topic is only raised in the spirit of dentists being warned against making unfair criticism against colleagues. The 2018 code places less emphasis upon dissuading professional criticism, but nevertheless fails to explicitly promote responsible self-regulation as a key professional obligation. This discourse is suggestive of a social reality where the dental profession places greater value upon the maintenance of harmony through avoiding the scrutiny of external parties to the profession, who might seek to attenuate the autonomy of practitioners. Given that self-regulation is given high importance within the social contract, this lack of reference would appear to be at odds with the obligations of the profession.

The 2018 Code of Conduct saw the addition of a section discussing the obligation of members of the ADA NSW to work to improve the oral health of the community, as well as that members should work to improve the standards, quality and access to dental services. These altruistic statements are accompanied by a statement enshrining the autonomy and independence of the dental profession. The social reality of dentistry in an environment where the majority of dentistry is provided privately finds the dental profession willing to support the concept of equity of access and community oral health, but not if this infringes on the self-determination of the dentists. Individual clinicians are limited in their responses to social issues [33]. Despite this, the profession collectively cannot be indifferent to injustice, inequality and the suffering caused by the structural issues created by the orientation of health services, insurers and commercial context of dental care.

The codes do not acknowledge another inequity that the dental profession has the ability to affect; the inequity that is intrinsic within the dentist-patient relationship. This is also likely reflected by the lack of community-input into the production of the ADA NSW Code of Ethics. The social contract that exists between the dental profession and society exists between all members of a profession. Being a member of the ADA NSW is voluntary; whilst the majority of those who practice in NSW are members, no judgement of a lesser standard of practice or ethics could be attributed to those who choose not to be. The Association should therefore be mindful of using the term “Member” as the descriptor; the idea that the Code of Ethics applies across the profession in a non-uniform manner should not matter if the purpose of the code is to give a set of aspirations that practitioners of the dental profession might be able to integrate into their own moral identity. The Code of Ethics should acknowledge that the granting of privilege and power to the dental profession is conditional upon practitioners showing willingness to conduct themselves in a manner that is consistent with wider social values. One way of acknowledging this and ensuring the voice of the public is present would be for representatives of the public to be involved in the next revision of the Code of Ethics. Whilst this process remains independent from wider society, the code will be unlikely to capture and be sensitive to the needs of the public and will fail to reflect the values the public that the profession has committed to serve.

## Conclusion

Through this critical discourse analysis, the codes of ethics published by the ADA NSW morph from being a declaration of commitments and values, to become a public record of the ideologies and power relations of the dental profession. The 2018 Code of Ethics portrays a dental profession that is more aware of its position of privilege in comparison to the profession that authored the 2012 incarnation. Within that awareness, the 2018 code would appear to demonstrate a sensitivity to acknowledge the origins of professional prestige gifted by society as part of the social contract. Whilst there have been developments within the 2018 Code of Ethics that demonstrate significant evolution in the social and professional attitudes of the ADA NSW, there is a crucial need for action to re-orient the Code of Ethics to include the voice of the public.

While this analysis is of two regional codes, the wider message of this research is pertinent to all professional associations. Codes of ethics created by professions that espouse duties to the public, but at heart demonstrate no commitments other than a profession’s own self-interest, are defunct due to breaching the social contract. Future editions of codes of ethics, or de novo versions, must demonstrate that the public have been involved in their creation. Proclamations of ethical values and positions made solely through consultation with the profession (or worse, a very select and small group of the profession), risk having a narrow perspective of what the public expects of the profession in twenty-first Century practice.

## Data Availability

Not applicable.

## Abbreviation

ADA NSW: Australian Dental Association, New South Wales Branch
